# Pilot and feasibility trials in traditional Chinese medicine: a literature review of current practice

**DOI:** 10.1186/s40814-020-00602-4

**Published:** 2020-04-22

**Authors:** Guowei Li, Darong Wu, Xuejiao Chen, Jie Zeng, Ziyi Li, Lehana Thabane

**Affiliations:** 1 Center for Clinical Epidemiology and Methodology (CCEM), Guangdong Second Provincial General Hospital, 466 Newport Middle Road, Haizhu District, Guangzhou, 510317 Guangdong Province China; 2 Department of Health research methods, Evidence, and Impact (HEI), McMaster University, 1280 Main St West, Hamilton, ON L8S 4 L8 Canada; 3 State Key Laboratory of Dampness Syndrome of Chinese Medicine, The Second Affiliated Hospital of Guangzhou University of Chinese Medicine, Guangzhou, China; 4 Guangdong Provincial Key Laboratory of Clinical Research on Traditional Chinese Medicine Syndrome, Guangzhou, China

**Keywords:** Traditional Chinese medicine, Pilot trial, Feasibility, Guideline adherence

## Abstract

**Background:**

The guidelines for pilot and feasibility studies were published in 2016. Little is known about the guideline adherence of TCM (traditional Chinese medicine) pilot trials or whether the guidelines can significantly enhance the quality of implementation and reporting of TCM pilot trials. We aimed to investigate the guideline adherence, assess the impact of guidelines on TCM pilot trials, and discuss potential challenges specific to TCM pilot trials, by conducting a literature review.

**Methods:**

We systematically searched MEDLINE, EMBASE, and CNKI to retrieve TCM pilot trials. We randomly chose 50 pilot trials from the eligible studies for analyses. The CONSORT extension to pilot and feasibility studies was used as a framework to assess the methodology and reporting quality of the studies.

**Results:**

The included studies had a guideline adherence level ranging from 4 to 96%, where the lowest adherence was found in the item 6c (prespecified criteria used to judge progression to future definitive trial). The guidance published in 2016 seemed to exert minimal effect on guideline adherence in TCM pilot trials. The unidentified issues related to TCM pilot trials from the guidelines included blinding, lack of standard formula of interventions, difficulty in comparison for effect assessment of interventions, and difficulty in bias control.

**Conclusions:**

The current practice in TCM pilot trials required substantial improvement in the literature. Further endeavors are needed for training and dissemination of guideline adherence, and development of more detailed methodology in the field of TCM pilot trials.

## Introduction

Pilot and feasibility trials have been published with a growing number. Pilot trials are significantly important for the design of a future main trial (or definitive trial) by providing evidence of feasibility issues and avoiding wasted recourses [1]. In 2016, Eldridge et al. published two critical publications aiming to reduce the misunderstanding and improve the reporting quality of pilot trials: the first providing a conceptual framework to define a pilot trial [2], and the second developing a CONSORT (Consolidated Standards of Reporting Trials) extension for pilot trials with a 26-item checklist included [3]. While the two publications may help with the design, implementation, reporting, and dissemination of pilot trials, it remains largely unknown about their impact on the pilot trials published in the literature. Confusions remained in the pilot trials including their definitions and terms, purpose, sample size determination, and criteria for progression or cessation, to mention a few [4–6].

Traditional Chinese medicine (TCM) is a hot topic in the health research community, especially given its alternative and integrated effect as a palliative treatment option [7]. Notably, some uncertainties and challenges exist in clinical trials for TCM that mainly include the difficulty in standardized procedures, potential heterogeneity in interventions and operators, control selection, and outcome assessment. Pilot trials for TCM offer a platform to identify and address these issues before a main trial. However, current evidence about the conduct and reporting of pilot trials for TCM is limited and sparse. Furthermore, little is known about whether the CONSORT extension for pilot trials can significantly enhance the quality of implementation and reporting of TCM pilot trials. Likewise, further evidence is needed to reveal the unidentified issues specific to TCM pilot trials from the guidelines [3]. Therefore, in this study, we conducted a literature review to investigate the guideline adherence of pilot trials for TCM, aiming to appraise the issues related to methodology and reporting. We also aimed to assess the impact of the CONSORT extension for pilot trials, and discuss any potential challenges specific to TCM pilot trials.

## Methods

### Search strategy and study selection

We systematically searched MEDLINE, EMBASE, and CNKI to retrieve TCM pilot trials. Descriptors including synonyms for traditional Chinese medicine or herbal medicine or folk medicine, and pilot trials or feasibility studies, were used in combination for the literature search (Supplemental Table 1 presents the search terms used). Studies were eligible for inclusion if they explicitly identified their TCM research as a randomized pilot or feasibility trial in the titles, abstracts, or introductions. Studies were excluded if they were not identified as a randomized pilot or feasibility trial, or they were not related to TCM, or they did not have information for methodological and reporting appraisal. Two reviewers (GL and XC) independently screened the records and determined study eligibility.

### Data extraction

Data extraction was completed by two independent reviewers (GL and XC). We categorized the included TCM pilot trials into two groups: (1) pilot trials that had at least one objective or assessment of feasibility and were conducted in preparation for a future definitive trial (FDT) and (2) trials that did not have feasibility objectives or assessment, termed as non-feasibility trials (NFT). This methodology was similar to Horne’s approach [8]. We assessed the guideline adherence about Title and Abstract (1a and 1b listed in the checklist), Introduction (2a and 2b), Methods (3a, 4c, 6a, 6c, 7a, and 12a), Results (13a), and Discussion (20, 21, and 22a) [3], separated by the two groups (FDT and NFT).

To document the methodological issues specific to TCM pilot trials, we also extracted the relevant data throughout the text from the included studies, especially in their Discussion sections.

### Statistical analyses

**Fig. 1:**
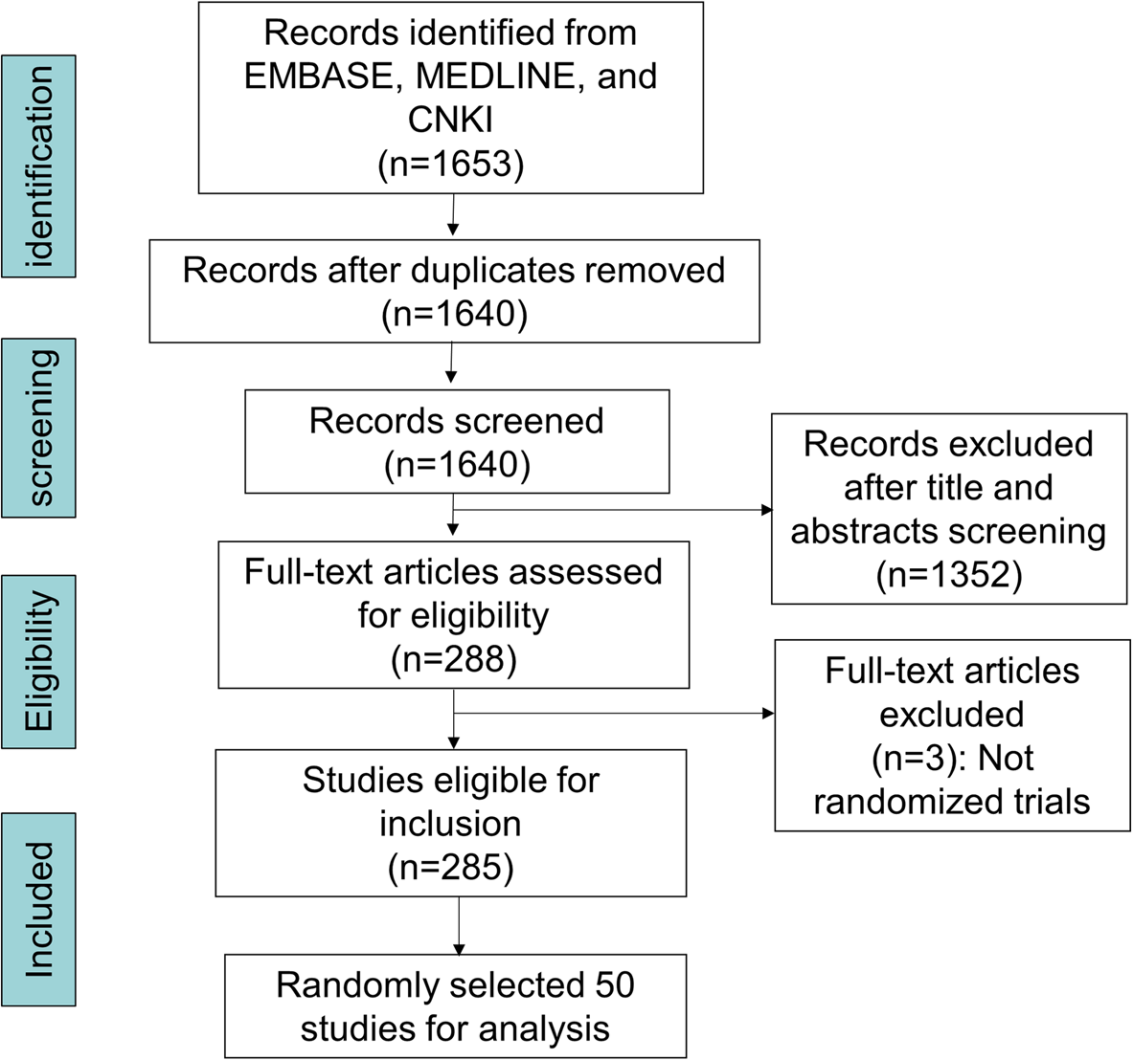
Flow diagram showing the process of eligible study identification

We expected that the proportion of FDT in our included studies would be approximately 15%. Therefore, we randomly chose 50 pilot trials from the 285 eligible studies for analyses (Fig. 1 shows the process of identifying eligible studies). To assess the impact of CONSORT extension for pilot trials on reporting, we selected the 50 studies that were published in either before or after the year 2016; i.e., no studies published in 2016 were identified for our analyses.

Guideline adherence was presented using counts and percentages. We performed a chi-square test to compare the guideline adherence levels between the two groups (FDT and NFT). To evaluate the impact of the CONSORT extension for pilot trials, we compared the guideline adherence of the included pilot trials published before and after 2016. When there was a cell with expected frequency < 5 in the contingency table, we used Fisher’s exact test to compare the guideline adherence levels between the groups. All analyses were conducted using the STATA version 13 (Stata Corp., College Station, TX, USA).

## Results

As shown in Fig. 1, we identified 285 eligible TCM pilot trials, among which 50 were randomly selected for analyses [9–58]. The selected 50 trials were published between year 1998 and 2019, and had a sample size ranging from 7 to 160 (Table 1). The TCM assessed in the trials included herbs, acupuncture, Chinese patent medicine, Qigong, massage, and others. There were 12 trials categorized as FDT (24%) and 38 as NFT (76%). Thirty-eight trials (76%) were published before year 2016, and 12 trials (24%) after 2016.

**Table 1:** Characteristics of the 50 included studies

First study author	Publication year	Journal	Country	Type of TCM	Number of participants randomized	Type of pilot trial
Agarwal	2014	*Asian Journal of Pharmaceutical and Clinical Research*	India	Herb	62	NFT
Ahn	2007	*Acupuncture in Medicine*	USA	Acupuncture	32	FDT
Avis	2008	*The Journal of The North American Menopause Society*	USA	Acupuncture	104	NFT
Chen	2003	*Maturitas*	China	Herb	44	FDT
Choi	2012	*The Journal of Alternative and Complementary Medicine*	Korea	Herb	40	NFT
Chung	2012	*Journal of Affective Disorders*	China	Acupuncture	50	FDT
Gong	2019	*Evidence-Based Complementary and Alternative Medicine*	China	Herb	63	NFT
Hsu	2008	*Advance Access Publication*	China	Herb	24	NFT
Huang	2019	*Plos One*	China	Herb	60	FDT
Iwasaki	2007	*Journal of the American Geriatrics Society*	Japan	Herb	48	NFT
Jones	2001	*BMC Complementary and Alternative Medicine*	China	Qigong	117	NFT
Kainuma	2004	*Human Psychopharmacology*	Japan	Herb	33	NFT
Kalman	2007	*Nutrition Journal*	USA	Chinese patent medicine	60	NFT
Kampman	2003	*Addictive Behaviors*	USA	Herb	14	NFT
Kang	1999	*Hong Kong Medical Journal*	China	Chinese patent medicine	120	NFT
Kong	2009	*Cerebrovasc Diseases*	Singapore	Herb	60	FDT
Kuo	2012	*Evidence-Based Complementary and Alternative Medicine*	China	Herb	28	NFT
Kuratsune	2010	*Phytomedicine*	Japan	Herb	12	NFT
Ladas	2010	*Cancer*	USA	Herb	106	FDT
Lee	2010	*Complementary Therapies in Medicine*	China	Herb	28	NFT
Lee	2011	*Planta Medica*	Korea	Chinese patent medicine	40	NFT
Li	2009	*Complementary Therapies in Medicine*	China	Herb	24	NFT
Li	2015	*HIV Clinical Trials*	China	Herb	140	NFT
Liew	2015	*Asia Pacific allergy*	Singapore	Chinese patent medicine	44	FDT
Liu	2018	*Evidence-Based Complementary and Alternative Medicine*	China	Chinese patent medicine	20	NFT
Luo	2018	*European Journal of Integrative Medicine*	China	Acupuncture	20	FDT
Noorbala	2005	*Journal of Ethnopharmacology*	Iran	Herb	88	NFT
Otto	1998	*American Academy of Addiction Psychiatry*	USA	Acupuncture	19	NFT
Pan	2018	*Chinese Journal of Integrative Medicine*	China	Other	60	NFT
Reshef	2013	*Sleep Disorders*	Israel	Acupuncture	27	NFT
Ritenbaugh	2008	*The Journal of Alternative and Complementary Medicine*	USA	Other	18	FDT
Scheid	2015	*Maturitas*	United Kingdom	Herb and/or acupuncture	42	FDT
Shelmadine	2017	*The Journal of Alternative and Complementary Medicine*	USA	Chinese patent medicine	56	NFT
Singh	2010	*Indian Journal of Medical Sciences*	India	Herb	7	NFT
Sitzia	2019	*Clinical Trial*	Italy	Other	56	NFT
Sordi	2019	*Journal of Natural Remedies*	Brazil	Herb	70	NFT
Spasov	2000	*Phytomedicine*	Russia	herb	128	NFT
Stockert	2007	*Pediatr Allergy Immunol*	Austria	Acupuncture	12	NFT
Tao	2013	*Evidence-Based Complementary and Alternative Medicine*	France	Other	40	NFT
Tsai	2018	*Complementary Therapies in Medicine*	China	Herb	160	NFT
Wang	2014	*Prev Chronic Dis*	USA	Herb and/or acupuncture	70	FDT
Wei	2015	*International Journal of Clinical and Experimental Medicine*	China	Chinese patent medicine	18	NFT
Wong	2006	*Journal of Child Neurology*	China	Acupuncture	120	NFT
Wu	2014	*Journal of Clinical Medical*	China	Acupuncture and massage	36	NFT
Wu	2015	*Neuropsychiatric Disease and Treatment*	China	Herb	46	NFT
Xu	2009	*Phytotherapy Research*	China	Chinese patent medicine	30	NFT
Yu	2018	*Journal of Acupuncture and Meridian Studies*	Canada	Acupuncture	60	NFT
Zhang	2015	*Journal of Alzheimer*’*s Disease*	China	Chinese patent medicine	12	NFT
Zou	2017	*Journal of Nutrition Health & Aging*	Canada	Other	21	FDT
Zou	2017	*Inquiry*	Canada	Other	36	NFT

*FDT* trials in preparation for a future definitive trial, *NFT* non-feasibility trials

Table 2 presents the detailed guideline adherence levels of the selected trials. The adherence ranged from 4 to 96%, with the lowest adherence found in 6c (prespecified criteria used to judge progression to future definitive trial) and highest in 12a (qualitative or quantitative methods used to address objectives). The checklist items 2b (specific objectives or research questions), 7a (rationale for sample size), and 21 (generalizability of methods and findings) also had low guideline adherence levels (18%, 8%, and 18% respectively). Table 2 also shows comparisons between FDT and NFT, and between studies published before and after year 2016. Compared with the NFT, the FDT had a significantly higher guideline adherence in the item 7a (rationale for sample size; 25% vs 3%) and 20 (discussion of study limitation, bias and uncertainty; 58% vs 34%). Guideline adherence level was only found significantly higher in the item 12a (qualitative or quantitative methods used to address objectives) in trials published after year 2016, when compared with studies published before 2016 (100% vs 55%).

**Table 2:** Details for guideline adherence of the included studies

Number of item	Checklist item	Guideline adherence				
		Overall studies (*n* = 50)	Subgroups^#^			
			By type of pilot trial		By year of publication	
			FDT (*n* = 12)	NFT (*n* = 38)	Studies published before 2016 (*n* = 38)	Studies published after 2016 (*n* = 12)
*Title and abstract*						
1a	Identification as a pilot or feasibility randomized trial in the title	47 (94.0)	11 (91.7)	36 (94.7)	36 (94.7)	11 (91.7)
1b	Structured summary of pilot trial design, methods, results, and conclusions (for specific guidance see CONSORT abstract extension for pilot trials)	37 (74.0)	9 (75.0)	28 (73.7)	27 (71.1)	10 (83.3)
*Introduction*						
2a	Scientific background and explanation of rationale for future definitive trial, and reasons for randomized pilot trial	11 (22.0)	3 (25.0)	8 (21.1)	8 (21.1)	3 (25.0)
2b	Specific objectives or research questions for pilot trial	9 (18.0)	3 (25.0)	6 (18.4)	8 (15.8)	1 (8.3)
*Methods*						
3a	Description of pilot trial design (such as parallel, factorial) including allocation ratio	35 (70.0)	8 (66.7)	27 (71.1)	26 (68.4)	9 (75.0)
4c	How participants were identified and consented	39 (78.0)	9 (75.0)	30 (79.0)	29 (76.3)	10 (83.3)
5	The interventions for each group with sufficient details to allow replication, including how and when they were actually administered	44 (88.0)	10 (83.3)	34 (89.5)	34 (89.5)	10 (83.3)
6a	Completely defined prespecified assessments or measurements to address each pilot trial objective specified in 2b, including how and when they were assessed	44 (88.0)	10 (83.3)	34 (89.5)	34 (89.5)	10 (83.3)
6c	If applicable, prespecified criteria used to judge whether, or how, to proceed with future definitive trial	2 (4.0)	1 (8.3)	1 (2.6)	1 (2.6)	1 (8.3)
7a	Rationale for numbers in the pilot trial	4 (8.0)	3 (25.0)*	1 (2.6)*	3 (7.9)	1 (8.3)
12a	Methods used to address each pilot trial objective whether qualitative or quantitative	48 (96.0)	11 (91.7)	37 (97.3)	21 (55.3)*	12 (100.0)*
*Results*						
13a	For each group, the numbers of participants who were approached and/or assessed for eligibility, randomly assigned, received intended treatment, and were assessed for each objective	34 (68.0)	10 (83.3)	24 (63.2)	26 (68.4)	8 (66.7)
*Discussion*						
20	Pilot trial limitations, addressing sources of potential bias, and remaining uncertainty about feasibility	33 (66.0)	7 (58.3)*	13 (34.2)*	27 (71.1)	6 (50.0)
21	Generalizability (applicability) of pilot trial methods and findings to future definitive trial and other studies	9 (18.0)	3 (25.0)	6 (15.8)	7 (18.4)	2 (16.7)
22a	Implications for progression from pilot to future definitive trial, including any proposed amendments	31 (62.0)	6 (50.0)	25 (65.8)	24 (63.2)	7 (58.3)

^#^two subgroup analyses conducted by study type (FDT vs NFT) and publication year (before 2016 vs after 2016)

*p value < 0.05 for difference test

The methodological issues specific to TCM pilot trials from the guidelines are shown in Table 3. There were 3 trials raising the issue of blinding in TCM pilot trials, mainly due to the acupuncture, administration forms, smells, and other reasons [12, 27, 51]. Other issues included lack of standard formula of interventions, difficulty in comparison for effect assessment of interventions, and difficulty in bias control [12, 27, 47, 58] (Table 3). For instance, in a pilot trial conducted by Choi et al., they reported that it was extremely difficult to evaluate the intervention effect because no standard treatment for atopic dermatitis could be used for comparison based on the current evidence-based TCM [58].

**Table 3:** Details of identified issues specific to TCM pilot trials

Issues specific to TCM pilot trials	Authors’ statements	Reference
Blinding; intervention	“in this study JWSYS [Jia-Wey Shiau-Yau San] was given in powder form and Premelle in tablet form. The question arises as to whether the women receiving JWSYS were aware that they were taking an established traditional Chinese herbal remedy. Since the trial was not a blind one and the improvement in the symptoms of these women could be due to an expectancy/placebo effect, given the cultural milieu”	Chen [12]
Randomization and blinding; intervention	“treatment with the complementary therapies of CM [Chinese medicine] had to be agreed by the patients or their families, thus randomly assigning the patients to the ST [standard treatment] or CH [Chinese herbs] by a completely blind method was difficult” “there was no fixed CM formula”	Lee [27]
Comparison and effect estimate	“in addition, there is no standard treatment for AD [atopic dermatitis] based on evidence-based medicine that could be used for comparison. Therefore, it is very difficult to rate an intervention compared to a standard herbal medicine”	Choi 2012 [58]
Blinding	“although the shape and color of the placebo were similar to Yueju, the smells of Yueju and placebo were not exactly identical, which may lead to the plausible incomplete blind treatment to patients.	Wu [51]
Intervention and bias control	“the current study cannot exclude the possible effects of HAT [herbal acupuncture therapy] on other factors, such as basic herbal regimens, proper acupuncture selection, and long-term therapeutic courses involved in the response of IDH [Intradialytic hypotension]”	Tsai [47]

## Discussion

In this study, we performed a review to assess the guideline adherence of TCM pilot trials. The guideline adherence varied crossing the checklist items, where some items required significant improvement. The guidance papers published in 2016 seemed to exert minimal effect on guideline adherence in TCM pilot trials. We also identified several issues specific to TCM pilot trials in this review including blinding, standards for intervention and comparisons, effect assessment, and bias reduction.

Interestingly, there were only 24% TCM pilot trials that had an objective of feasibility and were performed in preparation for future definitive trials (FDT). This indicated the inappropriate use of the term *pilot* in many small trials that aimed to test the hypotheses of efficacy or safety with an insufficient sample size albeit being underpowered to do so [8, 59, 60]. It also corresponded to the item 2b (specific objectives or research questions), where surprisingly only 3 (25%) in the FDT group clearly stated their objectives related to feasibility. Furthermore, there were only two items (7a and 20) found with significant improved guideline adherence in FDT compared with NFT, implying that more endeavors were required even in those pilot trials with specified feasibility objective(s). Therefore, all these findings suggested further dissemination of the guideline to help clarify the definition of feasibility and pilot trials [2] and to enhance the guideline adherence [3].

Likewise, our study indicated that the impact of CONSORT extension for pilot trials warranted more efforts in TCM pilot trials because the improvement was only found in one item (12a) after the guidelines were published (Table 2). The minimal effect of the guidance papers may be because either the guidelines did not reach the relevant research parties, or that the guidelines were largely ignored by the research parties [8]. In any case, our review reveals the urgent need for both training and dissemination of research methodology and guideline adherence in TCM pilot trials.

Besides the common practice of inappropriate hypothesis testing and insufficient power for conclusion in pilot trials [59, 61], our study also identified some issues specific to TCM pilot trials including blinding, standards for intervention and comparisons, and bias reduction (Table 3). This entails more guidance on methodology and reporting specific to TCM pilot trials because the existing guidelines including CONSORT extensions to acupuncture [62], herbal interventions [63], and pilot and feasibility studies [3] could not fully cover these issues in TCM pilot trials. The progression criteria (guideline adherence level, 4%), sample size rationale (18%), and generalizability of methods and findings (18%) were also notable issues found in the TCM pilot trials (Table 2). This may be, at least in part, due to insufficient details on explanation and elaboration from the guideline. For example, even though the CONSORT extension recommended that authors should justify the number of participants in pilot trials [3], no sufficient details on how to exactly provide sample size rationale could be found in the guideline. Likewise, how to specify the progression criteria to determine whether the pilot trial can progress to future main trial, and whether the methods and findings can be generalizable to main trial and other pilot studies, required further detailed investigation and guidance in TCM pilot trials. The TCM field is substantially different from modern medicine, especially in their intervention, control, and outcome assessment. For example, our review found that the issues specific to TCM pilot trials including blinding, standards for intervention and comparisons, effect assessment, and bias reduction, were not discussed in the CONSORT extension (Table 3). Thus, our findings call for the need for further methodology and guidance in the research area of pilot and feasibility studies to address the methodological issues and the other notable issues specific to TCM pilot trials.

Our study was the first to explore the current practice of methodology and reporting in TCM pilot trials. We completed the data acquisition and analyses by two reviewers independently, thereby enhancing the accuracy of study findings [64]. There are also some limitations to our study. Due to the small numbers of the included FDT (*n* = 12) and studies published after year 2016 (*n* = 12), we only performed raw comparisons without adjustments, which may yield biased findings in univariate analyses. We could not further extract potential solutions from the included TCM studies, indicating the important gap in methodological guidance in TCM pilot trials. Furthermore, only studies in Chinese and English were screened and selected, which may therefore introduce selection bias due to lack of studies in other languages such as Japanese and Korean. Moreover, the impact of time lag between the publication of a new guideline and the adoption and implementation of it could not be fully assessed, which may therefore weaken the findings of our study.

To conclude, the current practice in TCM pilot trials required substantial improvement in the literature. The guideline seemed to have only minimal effect on the methodology and reporting in TCM pilot trials, and some issues related to TCM pilot studies still warranted further methodology and guidance. Further endeavors are needed for training and dissemination of guideline adherence, and development of more detailed methodology in the field of TCM pilot trials.

## Supplementary information

**Additional file 1: Table S1**. Search terms used in the EMBASE, MEDLINE and CNKI.

## Data Availability

All the data are already publicly available in the literature.
