# Supplementary material for: Pilot and feasibility trials in traditional Chinese medicine: a literature review of current practice
Source: Pilot Feasibility Stud. 2020 Apr 22;6:48. doi: 10.1186/s40814-020-00602-4 (PMC7175575; doi:10.1186/s40814-020-00602-4)
Supplement: Supplementary file 1 — Additional file 1: Table S1. Search terms used in the EMBASE, MEDLINE and CNKI. [file 40814_2020_602_MOESM1_ESM.docx]

**Supplemental Table 1.** Search terms used in the EMBASE, MEDLINE and CNKI.

| **Steps** | **Search terms** |
| --- | --- |
| 1 | Chinese medicine |
| 2 | Traditional Chinese Medicine |
| 3 | Chinese herbal medicine |
| 4 | Chinese herbal drug |
| 5 | Traditional herbal medicine |
| 6 | Herbal medicine |
| 7 | traditional Chinese medical herb |
| 8 | folk medicine |
| 9 | botany |
| 10 | Chinese materia medica |
| 11 | phytotherapy |
| 12 | plant extract |
| 13 | medical plant |
| 14 | 1 or 2or 3 or 4 or 5 or 6 or 7 or 8 or 9 or 10 or 11 or 12 or 13 |
| 15 | pilot trial |
| 16 | feasibility study |
| 17 | pilot study |
| 18 | 15 or 16 or 17 |
| 19 | 14 and 18 |
